# Evaluation of Scrotal Pathologies by Ultrasound and Color Doppler

**DOI:** 10.7759/cureus.36776

**Published:** 2023-03-27

**Authors:** Kislay Kumar, Manisha Kumari, Vinod Kumar, Sanjay K Suman

**Affiliations:** 1 Radio Diagnosis, Indira Gandhi Institute of Medical Sciences (IGIMS), Patna, IND; 2 Radiology, Indira Gandhi Institute of Medical Sciences (IGIMS), Patna, IND

**Keywords:** color doppler ultrasound, color doppler, epididymis, testies, scrotal pathology, high resolution us

## Abstract

Introduction

Due to its ease of use, lack of ionizing radiation exposure, noninvasive nature, reproducibility, low cost, and ease of accessibility, ultrasound (US) is the preferred imaging modality for evaluating scrotal disease. High-resolution US and color Doppler better highlight scrotal and testicular diseases because of the scrotum's superficial anatomy. The genital organs are subjected to damaging ionizing radiation during CT, while MRI is both costly and uncommon.

Aims and objectives

The aim of this study is to use ultrasonography (USG) to examine various scrotal diseases and to diagnose and identify different disorders utilizing high-resolution US and color Doppler.

Materials and methods

The study was done on 60 patients who were referred to the Department of Radiodiagnosis by the General Surgery and Urology departments for the scrotal US and Doppler study. This study was conducted between October 2021 and March 2022 at the Indira Gandhi Institute of Medical Sciences, Patna, India.

Results

Out of 60 patients, hydrocele was seen in 25 cases, scrotal hernia in 12 cases, undescended testis in eight cases, varicocele and epididymal cysts in seven cases, etc. In acutely painful scrotal disorders, high-frequency US with color Doppler sonography successfully distinguishes between testicular ischemia/torsion and acute inflammatory illnesses. Eighteen cases of inflammatory scrotal pathologies and one case of testicular torsion were seen.

Conclusion

In detecting and assessing scrotal diseases, high-frequency USG and color Doppler sonography have good sensitivity and specificity. Furthermore, the lack of ionizing radiation, simplicity, wide availability, cost-efficiency, and reproducibility make it a highly important method for scrotal diseases.

## Introduction

The scrotum is a fibromuscular cutaneous bag that houses the testicles, epididymis, and the lowest half of the spermatic cord. Pathological conditions affecting these structures include congenital, simple inflammatory, and neoplastic diseases [1]. The first lines of imaging modalities for scrotal disorders include ultrasound (US) with color Doppler, magnetic resonance imaging, testicular angiography, and radioisotope studies. Patients who present with significant scrotal pain, edema, or both require prompt diagnosis in order to differentiate the lesions that require emergency surgical treatment from the lesions that may be treated medically [2]. The introduction of a sonogram with a high-frequency linear transducer and color Doppler is a significant step forward in assessing scrotal diseases. CT scans expose the testicles to radiation, and MRI is not widely accessible [3].

Though the testis is readily available for clinical examination, the acute scrotum is a tough diagnostic challenge due to the nonspecific character of symptoms and the difficulties in fully assessing the sensitive, swollen scrotum. The clinical history may be essential in determining the etiology of acute scrotal illnesses [4]. Although epididymitis is the most common cause of acute scrotal discomfort, testicular torsion is the most critical diagnosis to establish since it needs rapid surgical correction to maintain testicular viability and function. As a result, assessing testicular perfusion is the essential prerequisite for any imaging modality employed in this clinical setting [5].

Ultrasonography (USG) has been shown to be an excellent gold-standard diagnostic technique for a variety of scrotal diseases and disorders. It offers 100% sensitivity in diagnosis and can discriminate a range of disorders affecting the testis, epididymis, and scrotum with comparable clinical symptoms because of its great spatial resolution. USG in grayscale in conjunction with color or power Doppler imaging is a widely used tool for evaluating scrotal lesions and testicular perfusion. USG is an excellent tool for studying the scrotum and its contents. Sonography is simple to conduct, quick, noninvasive, affordable, easily repeatable, and generally available, and it does not need gonad irradiation. To identify testicular torsion, an emergency scrotal Doppler scan is sometimes necessary [6].

## Materials and methods

The study was done on 60 patients who presented to the general surgery and urology departments of Indira Gandhi Institute of Medical Sciences (IGIMS), Patna, India with pain, swelling, or clinically nonpalpable testis. After physical examinations, the patients were referred to the Department of Radiodiagnosis, IGIMS, for USG and color Doppler examination of the scrotum. This study was conducted between October 2021 and March 2022 at the IGIMS. Ethical committee permission was obtained from the hospital. Informed and written consent was taken from patients before examinations. Gray scale real-time imaging and color Doppler were done routinely.

Study design

The study was a hospital-based prospective observational study.

Statistical analysis

The data was transferred to a Microsoft Excel 2010 sheet (Microsoft Corporation, Redmond, Washington), and statistical analysis was done using SPSS (Statistical Package for the Social Sciences) software for Windows, version 22.0 (IBM Corp., Armonk, NY).

Equipment

US examination was performed using a SAMSUNG H60 ultrasound machine using an 8-MHz frequency linear probe.

Scanning technique

Scrotal USG is frequently conducted with the patient in a reclining position with their legs slightly spread, their scrotum supported by a rolled towel or pillow, and their penis wrapped in a towel and positioned on the abdominal wall. Scanning is normally done with a high-frequency transducer (8-MHz); however, an edematous scrotum requires a low-frequency transducer. Echogenicity, scrotal wall thickness, and flow symmetry are assessed by scanning the sagittal and transverse planes as well as transverse side-by-side planes of both testes. Additional methods, including standing and the Valsalva movement, were used to examine a varicocele. Color and power Doppler USG is used to detect perfusion issues and irregular flow patterns.

## Results

Table 1 shows the age distribution of cases. The highest number of cases presented was in the age group of 21-40 years (20 cases, 33.33%), followed by 41-60 years (13 cases, 21.67%). The mean age group of cases was 35.74 years.

**Table 1:** Age distribution

Age (years)	No. of cases	Percentage
0-10	9	15
11-20	7	11.67
21-40	20	33.33
41-60	13	21.67
>61	11	18.33

In our study, out of 60 cases, 18 cases were detected to have inflammatory scrotal pathology in the high-frequency US and Doppler studies (Table 2). Acute testicular inflammatory conditions generally show increased peak systolic velocity (PSV > 15 cm/s) and reduced resistance index (RI < 0.5). Acute epididymo-orchitis was the commonest inflammatory pathology detected, which was noted in five cases. The next most frequent inflammatory pathology detected was acute epididymitis, which was noted in four cases.

**Table 2:** Inflammatory pathology

Pathology	No. of cases	Percentage
Acute epididymo-orchitis	5	27.78
Acute epididymitis	4	22.22
Acute orchitis	1	5.56
Chronic epididymo-orchitis	1	5.56
Funiculitis	2	11.11
Cellulitis	3	16.67
Testicular abscess	1	5.56
Epididymal abscess	1	5.56
Total	18	

Among non-inflammatory scrotal swellings, hydrocele is the commonest pathology noted in 25 cases (35.21%) followed by scrotal hernia in 12 cases (16.90%), undescended testis in eight cases (11.26%), and epididymal cysts and varicocele in seven cases (9.86%) each (Table 3). Neoplastic testicular tumors, scrotal filariasis, testicular microcalcification, testicular torsion, etc. are other non-inflammatory scrotal pathologies. The incidence of non-neoplastic scrotal swellings is very much high compared to neoplastic swellings. The incidence of extra-testicular swellings is more compared to that of intra-testicular swellings.

**Table 3:** Non-inflammatory pathology

Pathology	No. of cases	Percentage
Hydrocele	25	35.21
Scrotal hernia	12	16.90
Undescended testis	8	11.26
Epididymal cyst	7	9.86
Varicocele	7	9.86
Neoplasia	3	4.22
Small testis	3	4.22
Scrotal filariasis	2	2.82
Testicular microcalcification	2	2.82
Testicular cyst	1	1.41
Spermatic cord hydrocele	1	1.41
Total	71	

**Figure 1:**
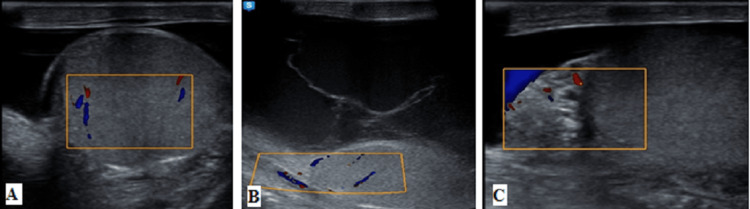
(A-C) Right acute epididymo-orchitis with infected hydrocele and scrotal wall cellulitis

 Right acute epididymo-orchitis with infected hydrocele and scrotal wall cellulitis is shown in Figure 1.

**Figure 2:**
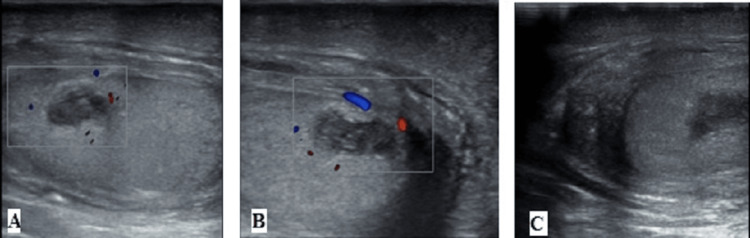
(A-C) Right testicular abscess with orchitis

The right testicular abscess with orchitis is shown in Figure 2.

**Figure 3:**
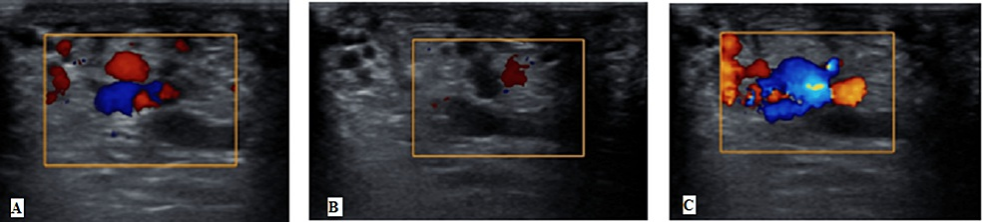
(A-C) Left-sided varicocele

**Figure 4:**
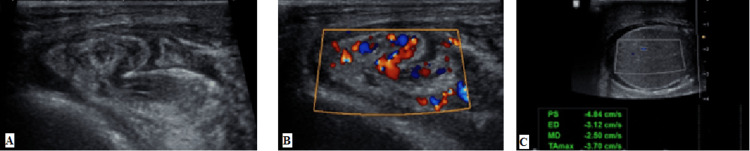
(A-C) Left testicular torsion

The left-sided varicocele and left testicular torsion are shown in Figures 3, 4.

## Discussion

The scrotal contents are appropriate for sonographic evaluation due to their superficial position. High-frequency real-time USG with color Doppler scanners has improved the diagnostic accuracy of scrotal sonographic tests. Scrotal USG has matured to the point that it can be used as the first and only imaging examination required to evaluate the scrotal contents.

Table 1 shows the age distribution of the patients in this investigation, which ranged from eight days to 80 years. The age group of 21-40 years had the most instances presented (20 cases, 33.33%), followed by 41-60 years (13 cases, 21.67%). In our investigation, hydrocele was the most prevalent disease seen in 25 out of 60 cases, followed by an inguinal hernia in 12 cases, undescended testis in eight instances, varicoceles in seven cases, epididymal cyst in seven cases, acute epididymo-orchitis in five cases, and scrotal neoplastic tumors in three cases. Gajbhiye et al. conducted prospective research with 200 patients, which was compatible with our results. The most prevalent disease in our analysis was hydrocele, which is consistent with the previous studies [7].

Vishnu et al. discovered that color Doppler sonography is 100% sensitive and 95% specific for the detection of testicular torsion [8]. When compared to physical examination, a research by Gajbhiye et al. reveals 100% sensitivity for color Doppler [7]. Doppler assessment was proven to be 100% sensitive and 100% specific for the detection of testicular torsion in a research by Middleton and Melson [9]. Burks et al.^ ^discovered that color Doppler sonography was 86% sensitive and 100% specific in the diagnosis of testicular torsion. This research looked at seven patients who had verified testicular torsion [10]. One case of testicular torsion was seen in our study, which showed absent blood flow and twisting of blood vessels on color Doppler, consistent with previous similar studies.

In our investigation, 18 out of 60 subjects were found to have inflammatory scrotal disease on high-frequency US and Doppler imaging. Acute epididymo-orchitis was the most prevalent inflammatory pathology seen, with five instances. Power Doppler analysis is a valuable tool in our research. It can be used to establish the lack of testicular perfusion in situations of suspected torsion as well as to improve traditional color Doppler results of hyperemia in testicular and epididymal inflammation. This is consistent with the findings of Farriol et al. and Luker et al. [11,12].^ ^Blood flow is seen in the typical adult epididymis when it is evaluated with a color and power Doppler. Only the asymmetric flow pattern in relation to the normal side might be used to diagnose epididymitis. This agreed with the findings of Keener et al. [13]. Our findings were compared to those of Petros et al. and Arger et al. [14,15]. Brown et al. indicated that if inflammation is suspected, spectrum analysis should be performed. They provided quantitative criteria for the diagnosis of inflammation, such as a PSV of 15 cm/s or higher in the epididymis or testis and a PSV ratio of more than 1.7 in the epididymis or 1.9 in the testis, which is equivalent to our study [16].

## Conclusions

Non-invasiveness, absence of ionizing radiation, simplicity, wide availability, cost-efficiency, and repeatability are all advantages of high-frequency US and color Doppler. High-frequency USG is quite sensitive in distinguishing between solid and cystic scrotal tumors. High-frequency real-time sonography is clearly superior to clinical diagnosis in identifying scrotal masses as testicular or extra-testicular masses. In the presence of big hydroceles, high-frequency USG is useful in confirming the normality of the testes and epididymis. The morphological changes associated with acute scrotal inflammatory disorders can be clearly seen by high-frequency USG. Color Doppler sonography is extremely sensitive in identifying acute scrotal illness and distinguishes testicular ischemia/torsion from acute inflammatory disorders. We conclude that high-frequency USG and color Doppler sonography are particularly useful tools in assessing scrotal and testicular diseases.
